# Perceived Helicopter Parenting and Korean Emerging Adults’ Psychological Adjustment: The Mediational Role of Parent–Child Affection and Pressure from Parental Career Expectations

**DOI:** 10.1007/s10826-018-1193-2

**Published:** 2018-07-21

**Authors:** Jaerim Lee, Sieun Kang

**Affiliations:** 1 Department of Child Development and Family Studies and the Research Institute of Human Ecology, Seoul National University, Seoul, Republic of Korea; 2 Department of Child Development and Family Studies, Seoul National University, 1 Gwanak-ro Gwanak-gu, Seoul, 08826 Republic of Korea

**Keywords:** Depressive symptoms, Helicopter parenting, Korean emerging adults, Life satisfaction, Parent–child relationships

## Abstract

Examining helicopter parenting in a Confucian culture that values parental authority and involvement can extend previous helicopter parenting research that has mostly focused on a U.S. college student context. In this study, we aim to help clarify the mixed results in the literature regarding the psychological outcomes of helicopter parenting in emerging adulthood by examining the processes underlying the relationship between helicopter parenting and psychological adjustment in the Korean context. Using a diverse sample of Korean emerging adults, we investigated the relationships among perceived helicopter parenting, parent–child affection, pressure from parental career expectations, and psychological adjustment (depressive symptoms and life satisfaction). The data came from 562 Korean emerging adults (269 women and 293 men) aged 19–34 years who were either full-time students or unemployed and unmarried and had at least one living parent. The structural equation modeling used in this study revealed that higher levels of helicopter parenting were directly associated with greater depressive symptoms. Higher levels of helicopter parenting also had an indirect relationship with greater depressive symptoms through higher levels of pressure from parental career expectations. However, higher levels of helicopter parenting were indirectly linked to better psychological adjustment (fewer depressive symptoms, greater satisfaction with life) through higher levels of parent–child affection. Our results indicate that in the East Asian context, helicopter parenting could be related to both negative and positive psychological outcomes depending on the mediating factors.

## Introduction

Helicopter parenting (hereafter HP) is a rapidly emerging topic in the literature. A growing number of researchers have studied how to conceptualize and measure HP (e.g., LeMoyne and Buchanan 2011; Padilla-Walker and Nelson 2012; Schiffrin et al. 2014; Segrin et al. 2012; Yoo and Jahng 2016) and how it is associated with child outcomes (e.g., Padilla-Walker and Nelson 2012; Schiffrin and Liss 2017; Schiffrin et al. 2014; Segrin et al. 2012). Although most studies have found a negative impact of HP, some studies have reported mixed results about the association between HP and psychological adjustment among emerging adults. Examining possible mediators may help resolve this inconsistency in the literature, especially in South Korea (Korea hereafter) where HP might play a unique role in parent–child relationships.

HP refers to overinvolved and overcontrolling parenting without giving the child sufficient autonomy to solve problems and make decisions (LeMoyne and Buchanan 2011; Schiffrin et al. 2014; Segrin et al. 2012). Since Cline and Fay (1990) first used the term “helicopter parent” in their book, the construct HP has been described as a type of overparenting (Bradley-Geist and Olson-Buchanan 2014; Segrin et al. 2012) and has gained empirical support as a distinct concept from behavioral and psychological parental control (see Padilla-Walker and Nelson 2012, for details). One of the reasons for the scholarly interest in HP is its unique combination of the important dimensions of parenting (e.g., support, control, autonomy granting) that are demonstrated in an excessive manner (Padilla-Walker and Nelson 2012; Rousseau and Scharf 2015). Although the popular press has often reported on issues related to HP for college students, this type of parenting begins prior to emerging adulthood (LeMoyne and Buchanan 2011), and the influence of HP often remains throughout emerging adulthood. In this study, we focus on perceived HP that many Korean emerging adults experience as they grow up. An investigation of perceived HP is appropriate to examine how HP is associated with a child’s adjustment through the parent–child relationship because it is the child’s perception of the parenting behavior rather than the parents’ intention behind the behavior that determines the role parenting plays in the child’s life (McKinney and Kwan 2018; Schiffrin and Liss 2017).

HP is a useful concept to explain parent–child relations in Confucian countries including Korea. Traditionally, Korean parents assumed the authority and responsibility to provide guidance and advice for their children even into adulthood. Based on a cultural emphasis on intergenerational interdependence and parental authority, it is still normative for parents to control and be actively involved in their children’s lives and decisions even after the children reach adulthood in Korea (Jang et al. 2016; Kwon et al. 2016). Parenting in Confucian cultures typically involves a simultaneous exercise of high control and intensive support (Leung and Shek 2018), two independent domains of parenting that constitute HP if they are combined to an excessive degree (Padilla-Walker and Nelson 2012; Rousseau and Scharf 2015). Thus, the possibility of practicing HP inherently exists in Confucian cultures.

As fertility rates drop in Korea, many Korean parents are motivated to devote an extensive amount of attention to and investment in a small number of children (Yoo 2014). Due to severe competition in the Korean educational system and labor market, anxious Korean parents provide intensive support for their children to alleviate the risks and difficulties that the children may face in the future (Chung 2014; Kim 2015), and in doing so, they often sacrifice their own needs for their children’s achievements. Through such support, these parents tend to maintain excessive control and decision-making power over their children (Kim 2015). Therefore, it is not surprising that many Korean parents are prone to use a HP style to overprotect their children from potential risks and maximize their children’s career opportunities.

Examining the role of HP is especially relevant for Korean emerging adults in their 20s and early 30s (Kang and Lee 2017) who are undergraduate and graduate students as well as those who have already graduated. Although Western researchers have commonly operationalized emerging adults as 18–29 years of age, the age range is not universal but is socio-culturally determined (Arnett 2015). The current cohort of Korean emerging adults is called the “2030 Generation,” referring to the generation in their 20 and 30s who share challenges in the labor market and demographic characteristics such as delayed marriage. The average age of the first marriage in Korea is 32.8 for men and 30.1 for women as of 2016 (Statistics Korea 2017c). Numerous college graduates in Korea remain unemployed for several years with the hope of better preparing for the labor market. Many of them are “kangaroo kids,” a popular term in Korea, because they are financially reliant on their parents and live in their parents’ homes until they marry (Chung 2014). Approximately 2 years of compulsory military service is another reason Korean men enter the labor force later compared to their peers in other countries (Park 2013). Thus, expanding the concept of emerging adults from college students to a broader group of emerging adults is necessary to understand the role of HP in the extended period of emerging adulthood in a Korean context (Kang and Lee 2017). U.S. scholars have also underscored the need to include non-college participants in emerging adulthood research (Schwartz 2016).

Whether and how HP is associated with emerging adults’ psychological adjustment has been a key area of interest in the literature on HP, but the results have been mixed. Most research has reported that HP is directly or indirectly linked to negative outcomes such as depressive symptoms and anxiety (Darlow et al. 2017; Kouros et al. 2017; Reed et al. 2016; Schiffrin et al. 2014) and lower life satisfaction (LeMoyne and Buchanan 2011; Reed et al. 2016; Schiffrin et al. 2014). Based on self-determination theory (Ryan and Deci 2000), HP is likely to be associated with maladjustment because this parenting style may violate the basic human needs for autonomy, competence, and relatedness (Schiffrin et al. 2014). Limiting children’s autonomy can be particularly harmful in adolescence and emerging adulthood when becoming autonomous is an important developmental task.

Some researchers, however, have found that HP has insignificant or even has positive associations with psychological adjustment and parent–child relationships (Fingerman et al. 2012; Kwon et al. 2016; Padilla-Walker and Nelson 2012). For example, Padilla-Walker and Nelson (2012) found that perceived HP among U.S. college students predicted better parent–child relationships in terms of higher levels of emotional support, disclosure, and parental guidance. In Fingerman et al.’s (2012) study, U.S. adult children (18–41 years) who received parental support several times per week or more frequently reported better psychological adjustment than grown children who did not receive such support. Using a sample of Korean college students, Kwon et al. (2016) found that HP was not directly related to Korean emerging adults’ emotional well-being. Despite the known negative impact of HP, some researchers have noted that HP could also play a positive role in some aspects of children’s psychological adjustment because the intention behind HP is benevolent and involves support for the child (Kwon et al. 2017; Padilla-Walker and Nelson 2012; Segrin et al. 2012).

The role of HP in psychological adjustment may have two sides in Korean culture. Based on Kagicibasi (2005) cultural model of autonomy and relatedness, they are two different dimensions. From the autonomy domain, HP is very likely to be associated with maladjustment. HP may lead children to be heteronomous (e.g., experiencing pressure and coercion: Regalia et al. 2013) because HP controls and limits children’s autonomy. From the relatedness domain, however, HP may not harm intergenerational relatedness because autonomy is not the opposite of relatedness but the opposite of independence (Kagicibasi 2005). Filial piety, a Confucian intergenerational value, provides an interesting cultural context in the two-sided role of HP. This cultural value requires children to treat their parents with respect, obedience, and devotion in appreciation of what the parents have done for them (Jorgensen et al. 2017; Yeh and Bedford 2003). On the negative side, HP may be associated with maladjustment due to its developmental inappropriateness and the stressful environment that HP creates. An example is the substantial burden that the children with helicopter parents feel when they believe they must to live up to their parents’ high expectations. Because filial piety socializes children to prioritize their parents’ expectations, pressure to fulfill parental expectations can be a major source of distress.

On the positive side, however, HP in Korean culture may be linked to intergenerational ties and consequently to better adjustment. Filial piety socializes children to unconditionally appreciate their parents’ involvement and control (Yoo and Liu 2014) regardless of how excessive it is. Thus, Korean emerging adults may value the benevolent intentions and supportive characteristics of HP (Kwon et al. 2017) unlike their counterparts in individualistic cultures who perceive HP as a serious threat to autonomy. For example, Korean emerging adults who have helicopter parents might believe that their parents are attempting to steer them in the right direction and are assisting with their academic achievements and career preparation. Similarly, in cultures that value relatedness, studies have shown that autonomy-limiting parenting styles have no negative impact on children’s adjustment (see Regalia et al. 2013, for details). Particularly given the extreme competition and challenges that emerging adults face in Korea, emerging adults may appreciate helicopter parents for providing a protective environment (Yoo and Jahng 2016). Thus, HP may be related to close relationships with the parents, which, in turn, may lead to better psychological adjustment.

An investigation of mediators in the relationship between HP and psychological adjustment can further our understanding of the mixed results in the literature. These mediators have been limited to individual characteristics such as autonomy, competence, and relatedness based on self-determination theory (Schiffrin et al. 2014), locus of control (Kwon et al. 2016), and self-efficacy (Bradley-Geist and Olson-Buchanan 2014; Reed et al. 2016). However, the characteristics of the parent–child relationship may also mediate the link between HP and psychological adjustment. Although independence from parents is an important developmental task in emerging adulthood, emerging adults’ relationships with their parents have a lasting impact on their psychological adjustment (Arnett 2015). In a culture that values filial piety, mediators related to parent–child relationships may be of particular interest in examining the effect of HP. In this study, we focus on two aspects of parent–child relations as potential mediators: parent–child affection and pressure from parental career expectations.

Parent–child affection, the emotional bond in the parent–child relationship, is one of the most vital aspects of parent–child relations in emerging adulthood (Arnett 2015; Roberts and Bengtson 1993). Based on the intergenerational solidarity model (Bengtson 2001), parent–child affection, the affectual dimension of intergenerational solidarity, represents feelings of emotional closeness, affirmation, and intimacy between parents and children (Roberts and Bengtson 1993). This dimension of intergenerational solidarity is considered the core of intergenerational relationships (Rossi and Rossi 1990). Unlike parental warmth or acceptance, which is a dimension of parenting, parent–child affection is a dimension of intergenerational relations.

Prior research has reported mixed results for the association between HP and the emotional aspects of parent–child relations among U.S. emerging adults. For example, Padilla-Walker and Nelson (2012) found that HP was positively associated with emotional support from parents, but Nelson et al. (2015) did not find significant correlations between HP and parental warmth. Instead, parental warmth moderated the relationship between HP and adjustment. Specifically, (Nelson et al. 2015) found that maternal HP was associated with better adjustment among emerging adults who experienced higher levels of maternal warmth, but among those who reported lower levels of maternal warmth, the association was in the opposite direction. Based on these results, the authors adjusted their earlier conceptualization of HP as being high parental warmth (Padilla-Walker and Nelson 2012) and suggested that HP is not inherently warm but is linked to maladjustment in the context of low warmth.

Affection is not necessarily an inherent characteristic of HP (Nelson et al. 2015), but in the Korean context, the levels of HP that emerging adults experienced while growing up may be positively associated with affection in their current parent–child relationship. Because Korean emerging adults tend to view intensive parental control and involvement as parental affection and efforts to support their children’s achievements (Jang et al. 2016; Song 2015), HP could help preserve affectionate parent–child relationships. In recent qualitative studies (Kang and Shih 2018; Kwon et al. 2017), Korean-American emerging adults perceived intensive instrumental support from their parents as a symbol of affection in a culture where verbal expression of love is rare. These emerging adults also stated that their perceptions of such parenting changed in a positive way as they became older and began to understand their parents’ sacrifice (Kang and Shih 2018). Thus, HP in the past might be recalled positively and contribute to parent–child affection in the present, which, in turn, would lead to better adjustment. The link between parent–child affection and psychological adjustment has been well established in the literature (Arnett 2015; Roberts and Bengtson 1993; Stafford et al. 2016).

Another potential mediator between HP and psychological adjustment is pressure from parental career expectations, which refers to children’s burden of attempting to live up to their parents’ expectations for a successful career. This perceived pressure stems from parents’ heavy emphasis on their children’s career success and could be stronger when their children perceive these expectations as being unrealistic for the child or as contradicting the child’s own wishes (Shim 2007). This emotional burden could be a result of HP. Because helicopter parents maintain high expectations and overly stress their children’s success, these children are likely to internalize the high expectations and become sensitive to whether they can fulfill these expectations, which creates emotional pressure. In particular, emerging adults in Confucian countries commonly prioritize their parents’ expectations over their own goals and wishes due to the influence of filial piety (Ma et al. 2018; Shin and Yoo 2012).

Pressure from parental *career* expectations is important in the extended period of emerging adulthood since helicopter parents are particularly involved in their children’s education and careers (LeMoyne and Buchanan 2011; Leung and Shek 2018). Whereas helping their children succeed in education is a focus before and during the children’s college years, a successful career is relevant throughout emerging adulthood. Concerns about their career is the most important matter among Korean emerging adults since obtaining a stable career is very challenging due to the heightened competition in the labor market. Many emerging adults are either unemployed or insecurely employed regardless of their education level. Nevertheless, most Korean parents maintain and express high expectations for their children’s careers (Chun and Lee 2014; Shim 2007) because a child’s career is not just an individual matter but a family matter. Occupational prestige determines social class in Confucian cultures, and children’s occupational achievements are considered a marker of the parents’ own success (Song 2015).

Due to filial piety, adolescents and emerging adults in East Asia tend to become vulnerable to negative emotions if they cannot fulfill their parents’ expectations, which are often very high (Oishi and Sullivan 2005). Particularly in emerging adulthood, pressure from parental career expectations may lead to lower levels of psychological adjustment. In a study conducted in Hong Kong (Ma et al. 2018), high parental expectations were associated with adolescents’ depression even though the high expectations contributed to better academic performance. Parents’ unrealistic expectations for their children’s career also play a negative role in Korean college students’ psychological adjustment (Jeong and Yoo 2015; Shin and Yoo 2012). Emerging adults who feel the burden of satisfying their parents’ career expectations may struggle with the fear of failing to secure a prestigious career and fulfill their parents’ wishes. In sum, emerging adults who perceive higher levels of HP are likely to be pressured to meet their parents’ expectations of securing a successful career, which can then lead to lower levels of psychological adjustment.

In this study, we aim to examine whether parent–child affection and pressure from parental career expectations mediate the relationship between HP and psychological adjustment. To do so, we examine a sample of unmarried Korean emerging adults aged 19–34 who are either full-time students or unemployed. Both the negative (i.e., depressive symptoms) and positive (i.e., life satisfaction) aspects of psychological adjustment are considered. We hypothesize that HP would be directly associated with depressive symptoms and life satisfaction (Hypothesis 1). We also hypothesize that HP would be indirectly related to depressive symptoms and life satisfaction through parent–child affection (Hypothesis 2) and through pressure from parental career expectations (Hypothesis 3). Given that unemployed young Koreans in their late 20s and early 30s remain reliant on their parents, we extend the age range of emerging adulthood beyond the traditional ages of college students.

## Methods

### Participants

We used a subset of data from a research project on emerging adults in Korea. The data were collected in April 2016 through a paid online survey service developed by a globally well-known research company. From the large online panel of the research company (approximately 1,500,000 Koreans), individuals who met three selection criteria were first filtered: (a) 19–34 years old, (b) never married, and (c) not in secondary education. This online panel was suitable to recruit a wide range of emerging adults with diverse backgrounds in Korea because approximately 60% of the panel was in their 20 or 30s with varied demographic characteristics. We had two sampling principles: (a) men and women had to be evenly distributed in each of the three age groups (19–24, 25–29, 30–34 years) and (b) the proportions of education levels, regions in Korea, employment status, and levels of parental income had to be similar to those of the entire Korean population aged 19–34. We initially aimed to collect data from 1000 individuals but ultimately used a larger sample size (1,148 respondents) to satisfy the sampling principles.

Given our focus on emerging adults who did not yet have careers, we narrowed our sample by selecting participants who were either full-time students or unemployed. We also chose emerging adults who had at least one living parent or parent-like figure due to our interest in HP and parent–child relations. The study sample consisted of 562 emerging adults (269 women and 293 men). The average age was 24.88 years (*SD* = 3.92), with 54.3% undergraduate college students, 27.4% college graduates, 8.9% graduate students, 8.5% high school graduates or lower, and 0.9% with a graduate degree. We believe that the education distribution of our participants is not biased because approximately 80% of Korean high school graduates attend college (Korea Education and Research Information Service 2017) and because most unemployed Koreans in their 20 and 30s are college graduates (Statistics Korea 2017d). Out of the 562 participants, 92% had two living parents (471 currently in a marital relationship, 55 divorced); however, 31 participants (5.5%) did not have a father, and 14 (2.5%) did not have a mother. Considering that Korean emerging adults in their 20s and early 30s have parents approximately 45–65 years of age, for whom bereavement rates are 2.7–14.8% (Statistics Korea 2016), the proportion of our participants with two living parents is similar to that of the entire Korean population in their 20s and early 30s.

Among the participants, 74.9% lived with at least one parent, which is a normative living arrangement for emerging adults in Korea. The parents’ monthly income was widely distributed, with 32.6% earning (in Korean currency) 2,000,000–3,990,000 KRW (approximately 1,900–3,800 US dollars) and 26.9% earning 4,000,000–5,990,000 KRW (approximately 3,800–5,700 US dollars). National statistics show that average household income is diverse depending on the householder’s age. As of 2016, national household income was 5,090,000 KRW for householders in their 50s and 2,910,000 KRW for those in their 60s or older (Statistics Korea 2017b). Most of the participants' fathers were either college graduates (39.9%) or high school graduates (34.5%), and high school graduates constituted the highest percentage of mothers (49.3%). These characteristics are similar to the national statistics. Among Koreans in their 50s, high school graduates were the most common (46.6%) and college graduates were the second largest group (28.5%) as of 2015 (Statistics Korea 2017a).

### Procedure

Emails were sent to invite randomly selected individuals who met the selection criteria. Among those who were invited, interested emerging adults accessed the survey website from their computers or smart devices. Once they provided informed consent and passed screening questions that confirmed their eligibility, they responded to the survey. The participants were not allowed to move to the subsequent set of questions if they skipped questions. When the questions were not relevant to the participant (e.g., not having a living father for the father’s HP questions), those questions were not shown. To incentivize participants to complete the entire survey, they received online points that could later be used in online or offline stores when accumulated.

### Measures

#### Helicopter parenting

HP was assessed by the Korean version of the Helicopter Parenting Scale (HPS; LeMoyne and Buchanan 2011). Participants were asked about the extent to which they perceived their mother and father as being overinvolved or overcontrolling as they grew up. This relatively comprehensive measure is applicable both to emerging adults who are college students and to those who are not college students and was validated among unmarried Koreans in their 20s and early 30s (Kang and Lee 2017). Other HP measures (e.g., Padilla-Walker and Nelson 2012; Schiffrin et al. 2014) are applicable only to college students because some items relate to college settings (e.g., schoolwork, grades, or classes). In addition, the retrospective nature of the HPS was appropriate for our mediational model hypothesizing that emerging adults’ *past* experiences with HP would shape *current* parent–child relationships, which, in turn, can make a difference in *current* psychological adjustment. In other words, this retrospective measure was useful to partially supplement the limitation of our cross-sectional data.

The HPS was developed for U.S. college students using ten items, but LeMoyne and Buchanan suggested deleting three items (#8, #9, #10) based on the results of their exploratory factor analysis (EFA). The Korean version of the HPS has been validated in a few studies with samples of Korean college students (Kwon et al. 2016; Yoo 2014; Yoo and Jahng 2016), and the validation was recently expanded to Korean young adults aged 19–34 by omitting three items (#3, #5, #10) based on the results of EFA and confirmatory factor analysis (CFA; Kang and Lee 2017). We used Kang and Lee’s version because we included older, non-student emerging adults.

HP was assessed separately for the mother and father. Sample items included, “My mother/father supervised my every move growing up,” “My mother/father often stepped in to solve life problems for me,” and “My mother/father has always been very involved in my activities” (1 = *strongly disagree*, 5 = *strongly agree*). A higher score indicated a higher level of perceived HP. We checked the validity and reliability of the Korean version of the HPS (Kang and Lee 2017) using EFA, CFA, and Cronbach’s alphas. The Korean HPS with seven items had a one-factor solution and fit the data adequately (mother: *χ*^2^ (14) = 67.976, *p* < 0.001, TLI = 0.911, CFI = 0.940, RMSEA = 0.084; father: *χ*^2^ (14) = 41.240, *p* < 0.001, TLI = 0.961, CFI = 0.974, RMSEA = 0.061). For structural equation modeling (SEM), we created two item parcels for each parent using an item-to-construct balance technique (Little et al. 2002); thus, we had four observed variables for a latent variable called HP. This approach was employed because a minimum of three to five observed variables is recommended for a latent variable (Kline 2015). Cronbach’s alphas for the seven items were 0.79 for mothers and 0.81 for fathers.

#### Parent–child affectionbetween the two aspects of psychological

Parent–child affection was measured by Roberts and Bengtson’s (1993) Perceived Parent-Child Affection Scale. We used the Korean version of this scale translated and validated by Kim and Lee (2015). The four items asked participants to evaluate how close their parent–child relationship was, how well they got along with their parents, how well they felt understood by their parents, and how well they understood their parents (1 = *not at all close/well*, 6 = *extremely close/well*). We used the same items for the mother and father separately, with higher scores indicating higher levels of affection for each parent. Likewise, we generated four item parcels, two for the mother and two for the father, for a latent variable called parent–child affection. Cronbach’s alphas for the four items were 0.91 for the mother and 0.93 for the father.

#### Pressure from parental career expectations

We assessed pressure from parental career expectations with a subscale of the Parental Career Expectations Scale (Shim 2007). This 18-item scale with four subscales (general, aptitude, family business, and economic expectations) was originally developed in Korean, and it has been widely used for Korean college students. We used the subscale of general career expectations, which measures the extent to which participants felt pressure from their parents’ career expectations and their perceived burden to meet their parents’ career expectations. The five items could be translated as (a) “It would be very hard to achieve the career plan that my parents set up for me,” (b) “My parents’ expectations for my career make it difficult to pursue my own career,” (c) “I feel pressured whenever I think of my parents’ expectations for my career,” (d) “It is hard to choose a career for my future because of my parents’ expectations,” and (e) “There is a big difference between my expectations and my parents’ expectations for my career.” A 5-point Likert scale was used (1 = *strongly disagree*, 5 = *strongly agree*), with higher scores indicating higher levels of perceived pressure. For SEM, we used the five items as five observed variables for a latent variable called pressure from parental career expectations. Cronbach’s alpha for the five items was 0.88.

#### Depressive symptoms

Depressive symptoms, one of the two indicators of psychological adjustment in this study, was assessed by the Center for Epidemiologic Studies—Depression Scale (CES-D; Radloff 1977). We used Lee’s (2002) Korean version of the CES-D. The 20 items asked participants to report how often they had felt each of the 20 depressive symptoms (e.g., feeling depressed, restless sleep, talking less than usual) in the past week (0 = *rarely or none of the time*, 1 = *some or a little of the time*, 2 = *occasionally or a moderate amount of the time*, 3 = *most or all of the time*). Although the original CES-D proposed four factors (depressed, somatic, positive, and interpersonal) for the 20 items, we used 16 items after deleting the positively worded items for two reasons. First, regarding the conventional use of the CES-D as a unidimensional measure, studies have reported an advantage of removing the four items related to positive emotions (Edwards et al. 2010; Stansbury et al. 2006). Second, the four items related to positive emotions were both conceptually and statistically correlated with life satisfaction, another indicator of psychological adjustment in this study. For SEM, we created four item parcels like we did for HP and parent–child affection. Because studies have reported inconsistent support for the original CES-D factor structure (Lee 2002; Stansbury et al. 2006), we chose item parceling instead of grouping items based on the original factors. The item parcels served as four observed variables for a latent variable called depressive symptoms. Cronbach’s alpha for the 16 items was 0.95.

#### Life satisfaction

Life satisfaction, another indicator of psychological adjustment, was assessed by the five-item Satisfaction with Life Scale (SWLS; Diener et al. 1985). The SWLS has been used frequently worldwide across a wide age range including Korean college students, because it measures the global judgment of one’s life rather than satisfaction with specific domains of life (Pavot and Diener 1993). We used Cho and Cha’s (1998) Korean version of the SWLS after modifying some wording to be more authentic to the meaning of the original scale in English. Sample items included, “In most ways my life is close to my ideal,” and “I am satisfied with my life” (1 = *strongly disagree*, 7 = *strongly agree*), with higher scores indicating higher levels of life satisfaction. We treated each of the five items as observed variables for a latent variable called life satisfaction. Cronbach’s alpha for the current study was 0.92.

### Data Analyses

We conducted structural equation modeling (SEM) using Mplus 8 to examine whether HP was associated with Korean emerging adults’ depressive symptoms and life satisfaction through parent–child affection and through pressure from parental career expectations. We used the full information maximum likelihood technique to handle the missing data for an absent parent and evaluated the model fit based on fit indices including the chi square, Tucker-Lewis index (TLI), comparative fit approximation (CFI), and root mean square error of approximation (RMSEA). TLI and CFI values of approximately 0.95 or greater indicate an excellent model fit (Hu and Bentler 1999), and RMSEA values of approximately 0.06 indicate a close model fit (Browne and Cudeck 1992).

Our SEM analysis involved testing the measurement model followed by the structural model. We first estimated the measurement model that included correlations among all five latent variables in the proposed model. This SEM analysis stage aimed to verify that the observed variables were properly mapped onto their latent constructs and that these latent variables co-varied with each other. After confirming that the measurement model fit our data, we estimated the structural model, which hypothesized all the pathways between our latent variables as well as the correlations between the two mediators and between the two aspects of psychological adjustment. To examine the significance of the indirect effects, we chose bootstrapping procedures with 5,000 bootstraps and a 95% confidence interval (CI; Shrout and Bolger 2002). If the 95% CI did not include zero, the mediating effect was considered significant. In our SEM analysis, we controlled for ebetween the two aspects of psychologicalmerging adults’ characteristics including gender, age, living with at least one parent, and attending college, as well as parents’ characteristics such as education and income. These control variables were chosen based on the Korean and U.S. literature suggesting that these characteristics might confound the associations of interest in this study (Bradley-Geist and Olson-Buchanan 2014; Chae et al. 2016; Kouros et al. 2017; Padilla-Walker and Nelson 2012; Schwartz 2016, Yoo 2014). Table 1 displays the correlational matrix and descriptive statistics for the observed variables.

**Table 1:** Intercorrelations and descriptive statistics (*N* = 562)

Variable	1.	2.	3.	4.	5.	6.	7.	8.	9.	10.	11.	12.	13.	14.	15.	16.	17.	18.	19.	20.	21.	22.
Helicopter parenting																						
1. Mother parcel 1^a^	–																					
2. Mother parcel 2^a^	0.70^***^	–																				
3. Father parcel 1^b^	0.52^***^	0.54^***^	–																			
4. Father parcel 2^b^	0.48^***^	0.63^***^	0.69^***^	–																		
Parent–child affection																						
5. Mother parcel 1^a^	0.28^***^	0.17^***^	0.19^***^	0.04	–																	
6. Mother parcel 2^a^	0.27^***^	0.18^***^	0.17^***^	0.01	0.88^***^	–																
7. Father parcel 1^b^	0.17^***^	0.13^**^	0.47^***^	0.28^***^	0.49^***^	0.47^***^	–															
8. Father parcel 2^b^	0.13^**^	0.14^**^	0.41^***^	0.24^***^	0.46^***^	0.46^***^	0.81^***^	–														
Pressure from parental career expectations																						
9. Item 1	0.22^***^	0.26^***^	0.22^***^	0.37^***^	−0.06	−0.07	−0.06	−0.01	–													
10. Item 2	0.22^***^	0.29^***^	0.25^***^	0.41^***^	−0.10^*^	−0.14^**^	−0.10^*^	−0.04	0.57^***^	–												
11. Item 3	0.19^***^	0.25^***^	0.21^**^	0.41^***^	−0.10^*^	−0.14^**^	−0.10^*^	−0.06	0.55^***^	0.67^***^	–											
12. Item 4	0.23^***^	0.31^***^	0.29^***^	0.42^***^	−0.11^*^	−0.15^**^	−0.05	−0.05	0.51^***^	0.70^***^	0.66^***^	–										
13. Item 5	0.17^***^	0.26^***^	0.26^***^	0.40^***^	−0.17^***^	−0.21^***^	−0.08	−0.06	0.51^***^	0.62^***^	0.60^***^	0.65^***^	–									
Depressive symptoms																						
14. Parcel 1	0.10^*^	0.23^***^	0.14^**^	0.23^***^	−0.20^***^	−0.17^***^	−0.11^*^	−0.10^*^	0.25^***^	0.27^***^	0.34^***^	0.32^***^	0.29^***^	–								
15. Parcel 2	0.13^**^	0.26^***^	0.16^***^	0.26^***^	−0.21^***^	−0.22^***^	−0.10	−0.10^*^	0.20^***^	0.27^***^	0.33^***^	0.34^***^	0.28^***^	0.87^***^	–							
16. Parcel 3	0.09^*^	0.25^***^	0.10^*^	0.23^***^	−0.24^***^	−0.23^***^	−0.11^**^	−0.10^*^	0.25^***^	0.28^***^	0.34^***^	0.32^***^	0.29^***^	0.85^***^	0.81^***^	–						
17. Parcel 4	0.11^**^	0.25^***^	0.07	0.20^***^	−0.24^***^	−0.24^***^	−0.16^***^	−0.14^**^	0.24^***^	0.26^***^	0.35^***^	0.30^***^	0.29^***^	0.80^***^	0.78^***^	0.81^***^	–					
Life satisfaction																						
18. Item 1	0.21^***^	0.13^**^	0.25^***^	0.15^***^	0.33^***^	0.32^***^	0.41^***^	0.38^***^	−0.11^*^	−0.07	−0.11^*^	−0.01	−0.08^*^	−0.15^***^	−0.13^**^	−0.16^***^	−0.20^***^	–				
19. Item 2	0.15^***^	0.06	0.23^***^	0.10^*^	0.35^***^	0.31^***^	0.41^***^	0.39^***^	−0.10^*^	−0.08	−0.12^**^	−0.05	−0.10^*^	−0.27^***^	−0.24^***^	−0.26^***^	−0.28^***^	0.77^***^	–			
20. Item 3	0.12^**^	0.07	0.23^***^	0.10^*^	0.37^***^	0.31^***^	0.43^***^	0.38^***^	−0.09^*^	−0.09^*^	−0.16^***^	−0.05	−0.08	−0.22^***^	−0.20^***^	−0.23^***^	−0.26^***^	0.75^***^	0.78^***^	–		
21. Item 4	0.18^***^	0.12^**^	0.23^***^	0.15^***^	0.33^***^	0.29^***^	0.39^***^	0.34^***^	−0.10^*^	−0.03	−0.08	−0.00	−0.04	−0.14^**^	−0.13^**^	−0.13^**^	−0.17^***^	0.69^***^	0.70^***^	0.75^***^	–	
–22. Item 5	0.10^*^	0.11^**^	0.26^***^	0.16^***^	0.25^***^	0.24^***^	0.41^***^	0.34^***^	−0.08	−0.03	−0.09^*^	0.02	−0.03	−0.14^**^	−0.10^*^	−0.12^**^	−0.16^***^	0.62^***^	0.63^***^	0.70^***^	0.66^***^	–
	Mother HP		Father HP		Affection for mother		Affection for father		Pressure from parental career expectations					Depressive symptoms					Life satisfaction			
Possible range	1–5		1–5		1–6		1–7		1–5					0–3					1–7			
*M*	3.11		2.95		4.40		3.76		2.89					.97					3.94			
*SD*	.69		.72		1.07		1.24		.91					.73					1.33			

^a^Excluded 14 participants who did not have a living mother

^b^Excluded 31 participants who did not have a living father

*p < 0.05. **p < 0.01. ***p < 0.001

## Results

### Measurement Model

**Fig. 1:**
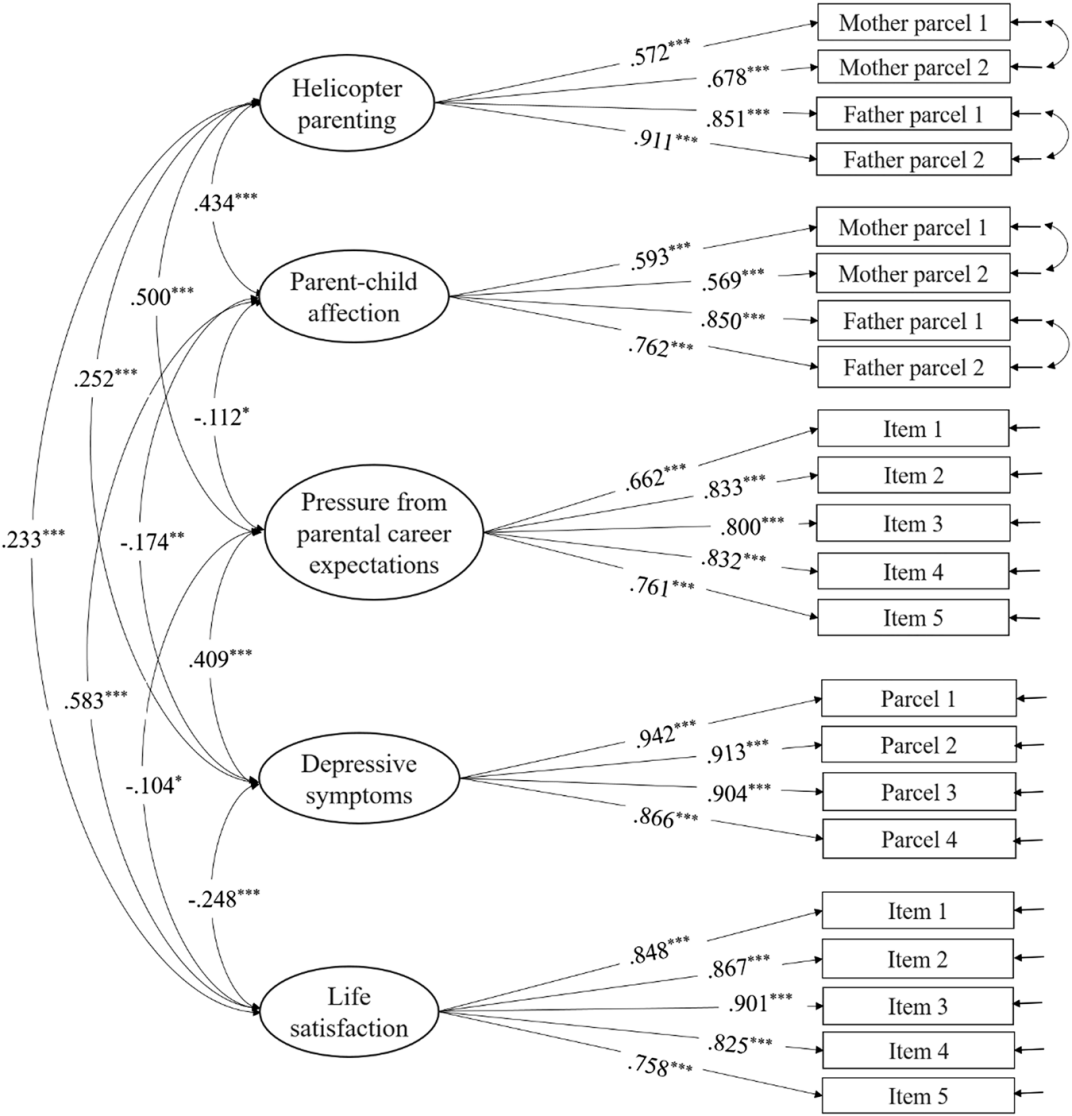
Measurement model: Standardized coefficients. Note: *χ*^2^ (195) = 549.266, *p* < 0.001; TLI = 0.954; CFI = 0.961; RMSEA = 0.051. *p < 0.05. **p < 0.01. ***p < 0.001

Figure 1 displays the results of the final measurement model, a slight modification of the initial measurement model. The modification indices of our initial measurement model suggested that the fit was reasonable but could be improved by including correlations between the residuals of item parcels for the same parent (i.e., two parcels of mother’s HP, two parcels of father’s HP, two parcels of mother-child affection, two parcels of father-child affection). We accepted these suggestions since it made sense to add these four correlations because these item parcels were generated from the same measures assessing the same constructs for the same parent. These correlations can be found in Fig. 1. The final measurement model had a good fit for the data, *χ*^2^ (195) = 549.266, *p* < 0.001, TLI = 0.954, CFI = 0.961, RMSEA = 0.051. The loadings of all observed variables were 0.569 or higher. Therefore, each of the observed variables in the measurement model was well mapped onto its latent variable, and it was appropriate to use the 22 observed variables to explain the five latent constructs.

### Structural Model

**Fig. 2:**
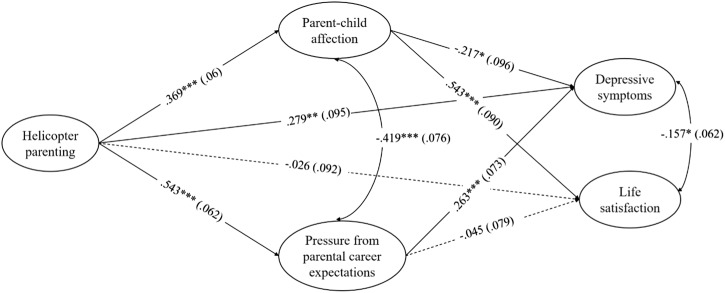
Structural model: Standardized coefficients and standard errors. Note. Standard errors are in parentheses. Dotted lines indicate non-significant paths. Controlling for children’s characteristics (gender, age, college student status, living with at least one parent) and parents’ characteristics (education, income) are not shown. *χ*^2^ (297) = 685.85, *p* < 0.001; TLI = 0.949; CFI = 0.958; RMSEA = 0.048. *p < 0.05. **p < 0.01. ***p < 0.001

Figure 2 displays the results of our structural model. The fit indices indicated a good fit between the proposed model and our data, *χ*^2^ (297) = 685.85, *p* < 0.001, TLI = 0.949, CFI = 0.958, RMSEA = 0.048. Table 2 displays the bias-corrected 95% bootstrap confidence intervals (CI) for direct, total indirect, specific indirect, and total effects in our structural model.

**Table 2:** Standardized bootstrap estimates and 95% bias-corrected confidence intervals for direct, indirect, and total effects

			BC 95% CI	
Effect	*β*	SE	CI_lower_	CI_upper_
Effects from HP to *depressive symptoms*				
Direct	0.279	0.095	0.113	0.485
Total indirect	0.063	0.071	−0.099	0.183
Specific indirect				
HP → Affection → Depressive symptoms	−0.080	0.039	−0.181	−0.019
HP → Pressure → Depressive symptoms	0.143	0.040	0.070	0.231
Total: Direct + Total indirect	0.342	0.056	0.234	0.454
Effects from HP to *life satisfaction*				
Direct	−0.026	0.092	−0.227	0.138
Total indirect	0.176	0.080	0.044	0.370
Specific indirect				
HP → Affection → Life satisfaction	0.201	0.049	0.124	0.320
HP → Pressure → Life satisfaction	−0.024	0.044	−0.108	0.066
Total: Direct + Total indirect	0.150	0.057	0.040	0.263

HP = helicopter parenting, affection = parent–child affection, pressure = pressure from parental career expectations

#### Relationships between HP and depressive symptoms

The direct association between HP and depressive symptoms was significant and positive (*β* = 0.279, *p* < 0.01. 95% CI = 0.113, 0.485). This indicates that emerging adults who perceived higher levels of HP reported greater depressive symptoms. The size of this direct effect was large (Kenny 2018). The association between HP and parent–child affection was significant (*β* = 0.369, *p* *<* 0.001), and parent–child affection was related to depressive symptoms (*β* = −0.217, *p* *<* 0.05). This means that emerging adults who experienced HP were more likely to report greater affection for their parents, which, in turn, led to fewer depressive symptoms. As shown in Table 2, the indirect effect from HP to depressive symptoms through parent–child affection was significant (*β* = −0.080, 95% CI = −0.181, −0.019), but the effect size of this indirect pathway was small.

We found a significant path between HP and pressure from parental career expectations (*β* = 0.543, *p* *<* 0.001) as well as the path between the pressure and depressive symptoms (*β* = 0.263, *p* *<* 0.001). Specifically, emerging adults who perceived higher levels of HP were more likely to feel pressure from parental career expectations, leading to greater depressive symptoms. The indirect effect through the pressure from parental career expectations (*β* = 0.143, 95% CI = 0.070, 0.231) was significant. Taking the two mediators together, the total indirect effect from HP to depressive symptoms through both mediators was not significant (*β* = 0.063, 95% CI = −0.099, 0.183) mainly due to the opposite direction of the two indirect effects. Finally, the total effect from HP to depressive symptoms, the sum of direct and total indirect effects, was significant (*β* = 0.342, 95% CI = 0.234, 0.454). The large total effect size is mainly due to the large size of the direct effect from HP and depressive symptoms.

#### Relationships between HP and life satisfaction

The direct relationship between HP and life satisfaction was not significant (*β* = −0.026, *ns*, 95% CI = −0.227, 0.138). However, we found an indirect association between HP and life satisfaction. Both the link between HP and parent–child affection (*β* = 0.369, *p* *<* 0.001) and the link between parent–child affection and life satisfaction (*β* = 0.543, *p* *<* 0.001) were significant. Specifically, emerging adults who perceived higher levels of HP were more likely to have greater affection for their parents, which was, in turn, related to higher life satisfaction. According to the bootstrapping result shown in Table 2, this indirect effect through parent–child affection was significant (*β* = 0.201, 95% CI = 0.124, 0.320).

The association between HP and pressure from parental career expectations was also significant (*β* = 0.543, *p* *<* 0.001), but pressure from parental career expectations was not related to life satisfaction. The bootstrapping result in Table 2 also indicated that the specific indirect effect through pressure from parental career expectations was not significant (*β* = −0.024, 95% CI = −0.108, 0.066). The total indirect effect from HP to life satisfaction through both parent–child affection and pressure from parental career expectations was significant (*β* = 0.176, 95% CI = 0.044, 0.370). For the total effect, a sum of direct and total indirect effects, HP was found to have a significant, positive total effect on life satisfaction (*β* = 0.150, 95% CI = 0.040, 0.263). However, this positive total effect was much smaller than the total negative effect from HP to depressive symptoms (*β* = 0.342, 95% CI = 0.234, 0.454).

## Discussion

In this study, we hypothesized that there would be a direct association between the HP that Korean emerging adults experienced as they grew up and their current psychological adjustment (i.e., depressive symptoms and life satisfaction; Hypothesis 1). We also hypothesized that there would be indirect relationships between HP and psychological adjustment through parent–child affection (Hypothesis 2) and through pressure from parental career expectations (Hypothesis 3). We conducted SEM using a sample of 562 Korean emerging adults who were 19–34 years old, were either full-time students or unemployed, unmarried, and had at least one living parent.

Hypothesis 1 was partially supported. Perceived levels of HP were directly associated with depressive symptoms among Korean emerging adults but were not directly related to life satisfaction. In other words, Korean emerging adults who perceived that their parents were overinvolved in their lives were more likely to experience depressive symptoms, and the size of this direct effect was large. This result is consistent with prior U.S. studies documenting a significant link between HP and depressive symptoms (Darlow et al. 2017; LeMoyne and Buchanan 2011; Schiffrin et al. 2014). These U.S. studies argued that the intrusive, controlling nature of HP appears to conflict with emerging adults’ desire for autonomy, and thus stimulates negative emotions such as depressive symptoms. Our finding supports Kagicibasi (2005) who suggested that becoming an autonomous self is important not only in individualistic countries but also in economically developed familistic cultures. Notably, our sample included both full-time students and unemployed emerging adults, and both groups typically receive a substantial amount of financial assistance from their parents in Korea. In fact, approximately 37% of our participants completely relied on their parents for their living expenses. Korean parents who provide financial assistance for their emerging adult children are likely to maintain control over their children (Kim 2015). Thus, emerging adults who are financially dependent on their helicopter parents may struggle emotionally due to difficulties making autonomous decisions without parental influence.

Unlike the link between HP and depressive symptoms, HP was not directly related to life satisfaction among Korean emerging adults. Therefore, the direct role of HP in overall perceptions of current lives was neither negative nor positive. This finding is not consistent with prior U.S. research that has reported a direct, negative impact of HP on emerging adults’ adjustment (Darlow et al. 2017; LeMoyne and Buchanan 2011; Padilla-Walker and Nelson 2012). However, the result is in line with Kwon et al. (2016), who found a non-significant direct association between HP and emotional well-being among Korean college students. In sum, the partial support for Hypothesis 1 implies that HP can lead to psychological distress for Korean emerging adults, but such parenting is not linked to the overall evaluation of their lives. The relationship between HP and life satisfaction seems to be complicated, which calls for further examination of the mediation between these two concepts.

The mediating effect of parent–child affection (Hypothesis 2) was supported for the indirect association between HP and life satisfaction as well as the indirect link between HP and depressive symptoms. Korean emerging adults who experienced HP as they grew up maintained more affectionate relationships with their parents, leading to fewer depressive symptoms and greater life satisfaction. This finding contradicts prior research warning that parental overinvolvement and overcontrol could hinder emerging adults from developing a secure attachment to their parents (Jeon 2015). Our result also contradicts the U.S. literature on HP. For example, Schiffrin et al. (2014) reported that HP had a negative link to U.S. college students’ sense of relatedness, one of the three basic psychological needs in self-determination theory. Segrin et al. (2012) found that overparenting was negatively associated with family satisfaction through parent-adolescent communication. In Nelson et al. (2015) study, parental warmth was not significantly correlated with HP but moderated the association between HP and adjustment.

The positive association between HP and parent–child affection in this study may be because autonomy and relatedness are two different domains as Kagicibasi (2005) posited. From this theoretical perspective, HP can deteriorate children’s independence by severely limiting autonomy but it may not harm their intergenerational relatedness, framed as parent–child affection in this paper. HP may be linked to parental guidance and emotional support (Padilla-Walker and Nelson 2012), which promote children’s emotional ties with their parents. In addition, this positive association may be because emerging adults perceive parental overinvolvement as attentive or supportive (Kwon et al. 2017; Somers and Settle 2010; Wartman 2009). It has been documented that, as emerging adults grow older, they tend to have improved parent–child relationships compared to adolescents (Arnett 2015) and tend to interpret intensive parental support in a more positive way (Kang and Shih 2018). Given the retrospective nature of our HP measure, Korean emerging adults may have come to realize the benevolent intention of the HP that they experienced in the past, which is likely to lead to greater parent–child affection in emerging adulthood.

The Korean context can further help us understand the mediational role of parent–child affection in this study. Under the cultural influence of filial piety that highlights children’s appreciation for parents, Korean or Korean-American emerging adults tend to regard intensive parenting as parental efforts to give them a better future or as a means of expressing love and attention for their children (Kang and Shih 2018; Kwon et al. 2016; Yoo and Jahng 2016). HP, an excessive type of intensive parenting, may help children feel loved and supported because of the perceived parental affection and benevolent intentions (Kwon et al. 2017; Yoo and Jahng 2016). When the culture of filial piety meets emerging adults’ harsh realities like severe competition at school and in the labor market, extreme parenting styles like HP may provide a psychological shelter through parent–child affection despite the potential negative effects of HP. Future research needs to investigate this speculation by examining cultural and societal factors. In addition, HP may include intensive financial support that can offer economic security to college students and unemployed emerging adults. Korean emerging adults who financially rely on their parents tend to remain emotionally close to their parents, which makes it easier or more comfortable for them to be content in a dependent relationship (Kim 2015). In this Korean context, HP can help emerging adults develop better parent–child relationships and eventually lead their children to experience better adjustment.

The mediational role of pressure from parental career expectations (Hypothesis 3) was partially supported for the association between HP and depressive symptoms. Korean emerging adults who perceived that their parents were overinvolved and overcontrolling were more likely to feel pressure from their parents’ career expectations, leading to greater depressive symptoms. This result suggests that when parents are overinvolved without allowing their children to experience sufficient autonomy to solve problems and make decisions, their emerging adult children are likely to feel the burden of satisfying their parents’ high career expectations. Korean emerging adults may be vulnerable to this burden because children in filial piety cultures tend to prioritize their parents’ expectations and are worried about disappointing their parents (Ma et al. 2018). The significant link between pressure from parental career expectations and greater depressive symptoms in this study is in line with Jeong and Yoo (2015). These authors noted that Korean college students who perceived higher levels of parental expectations for their future career experienced psychological distress in attempting to fulfill their parents’ high expectations.

Interestingly, the mediational role of pressure from parental career expectations was not supported when the psychological outcome was life satisfaction because pressure was not associated with life satisfaction. This result indicates that Korean emerging adults who felt greater pressure from their parents’ career expectations did not necessarily have a negative perception about their overall life even though they experienced emotional difficulties such as fear of not being able to fulfill their parents’ expectations. It is possible that pressure from high parental expectations as a result of HP may also be related to feeling like a special child for whom the parents maintain high expectations. This feeling may have confounded the negative effect of the pressure on their overall evaluation of life by prompting the child to strive for a better life. A study of adolescents in Hong Kong showed that high parental expectations were linked to better academic performance as well as greater depressive symptoms (Ma et al. 2018), which suggests that high parental expectations play a complex role. Helicopter parents’ high expectations could also lead to narcissism (Segrin et al. 2013). The literature has shown that narcissism can be associated with both adjustment and maladjustment (Pausen et al. 2016). Further, the mixed mediation effects of pressure from parental career expectations imply that psychological distress and the subjective status of life are different dimensions of psychological adjustment (Bryant and Veroff 1982), particularly among Korean emerging adults.

### Limitations

The present result should be interpreted in light of several limitations. As an early study of HP in Korea, we did not test within-group differences or moderation effects for the sake of parsimony. Demographic characteristics were simply controlled for in the present study. However, an examination of moderated mediation (e.g., a multiple group SEM) may have provided valuable empirical information. For instance, emerging adults’ characteristics such as gender, age, student status, or living arrangement along with parents’ characteristics such as gender and socioeconomic status might moderate the mediational pathways found in this study.

The moderators that are more specific to the Korean context may have been meaningful to explain the mixed relationships between HP and adjustment in this study. Because we did not have data on emerging adults’ attitudes toward HP, we speculated that Korean emerging adults might not be totally negative but somewhat ambivalent toward HP based on previous studies (e.g., Kwon et al. 2017; Yoo and Jahng 2016). Ideally, we would have measured the emerging adults’ attitudes toward HP and examined the moderating role in our structural model. For instance, among Korean emerging adults who perceive HP as a sign of parental affection, HP could be associated with better adjustment through better parent–child relationships. However, among those who view HP as an unhealthy parenting practice, HP may harm their well-being. Another potential moderator is how much Korean emerging adults endorse traditional cultural values such as filial piety, which may have a substantial influence on their attitudes toward parenting and parent–child relationships. Like Jorgensen et al. (2017) study reporting the moderation effect of Chinese college students’ filial piety in the relationships between perceived parenting practice and self-esteem, the role of HP might differ depending upon the Korean emerging adults’ sense of filial piety. HP might have a more negative impact on those with lower levels of filial piety because they may be more critical of HP. To better understand the complicated relationships between HP and psychological adjustment, future research should examine moderators such as emerging adults’ attitudes toward HP or their sense of filial piety.

Our structural model was limited to the mediators related to parent–child relationships, to which previous research has not paid adequate attention. However, other mediators may have confounded the negative effects of HP and pressure from parental career expectations on emerging adults’ life satisfaction in this study. We suggest that future researchers examine the mediators that may capture the unique context of Korean emerging adults’ lives. One example is the factors related to their career preparation such as college ranking, major, or GPAs along with career decision-making self-efficacy (Chun and Lee 2014). It would also be helpful for future researchers to consider emerging adults’ internal factors such as narcissism to explain the insignificant path between pressure from parental career expectations and life satisfaction.

Our measure of perceived HP, the Helicopter Parenting Scale (HPS: LeMoyne and Buchanan 2011), had some limitations. The HPS was appropriate to examine HP among Koreans in their 20s and early 30s (Kang and Lee 2017) compared to other measures that ask about HP behaviors, particularly those that are specific to the U.S. college context. Although we used cross-sectional data, the retrospective nature of the HPS was useful to explain the hypothetically causal relationship between HP and other study variables. However, retrospective measures are restricted because memories can be inaccurate and susceptible to the present circumstances. Parent–child affection in the present may have shaped how emerging adults perceived HP in the past, which is the opposite direction from our path model. In addition, the instructions of HPS did not provide a clear timeframe to reflect on other than “while growing up.” Since this timeframe is vague, some may have responded to the HPS in reference to a longer period of time (e.g., since childhood) while others may have interpreted the instruction as a shorter period of time (e.g., since adolescence). Another issue is whether the respondents were supposed to consider present parenting styles or not. Two items of the HPS are in the present perfect or present tense unlike the remaining items in the past tense. Although multiple studies have verified the validity of the HPS for Korean emerging adults (Kang and Lee 2017; Kwon et al. 2016), more research is needed to strengthen this measure.

Finally, the cross-sectional design of this study did not allow us to determine causality of the proposed relationships nor to investigate the long-term effect of HP. It is also possible that better psychological adjustment may lead to greater parent–child affection or lower pressure from parental expectations. Further, because we measured perceived HP only at one point in time, we do not know whether and how the levels of HP change over time as the child becomes older and how this change is associated with parent–child relationships and psychological adjustment. We could not investigate the longitudinal influence of HP, which is largely unknown in HP research. Longitudinal data would help to accurately identify the trajectories and consequences of HP.

Despite these limitations, this study advances our understanding of the roles of perceived HP in psychological adjustment among emerging adult in Confucian cultures. The present study revealed that HP was directly associated with greater depressive symptoms among Korean emerging adults. Depending on the mediators—different aspects of the parent–child relationship—we found that HP can be both positively and negatively related to emerging adults’ psychological adjustment. One of the contributions of this study is the focus on the overlooked mediators of parent–child relations. This study also extends the previous HP research that has been restricted to college students to a wider group of emerging adults.
